# Bridging fundamental RNA biology, retroviral replication, and oncogenesis: Karen Beemon wins the 2007 Retrovirology Prize

**DOI:** 10.1186/1742-4690-4-88

**Published:** 2007-12-10

**Authors:** Kathleen Boris-Lawrie

**Affiliations:** 1 Department of Veterinary Biosciences, Center for Retrovirus Research, The Ohio State University, 1925 Coffey Road, Columbus, OH 43210, USA

## Abstract

The 2007 M. Jeang *Retrovirology *Prize has been awarded to Dr. Karen L. Beemon

The *Retrovirology *Prize, awarded annually, recognizes an outstanding mid-career retrovirologist aged 45 to 60 [1,2]. The prize, supported by the Ming K. Jeang Foundation, alternates between HIV and non-HIV research. In 2005, Dr. Stephen P. Goff was the prize winner [3]; and last year, Dr. Joseph G. Sodroski was recognized for his HIV research [4]. The 2007 awardee of the *Retrovirology* prize is Dr. Karen L. Beemon  (Figure 1).

**Figure 1:**
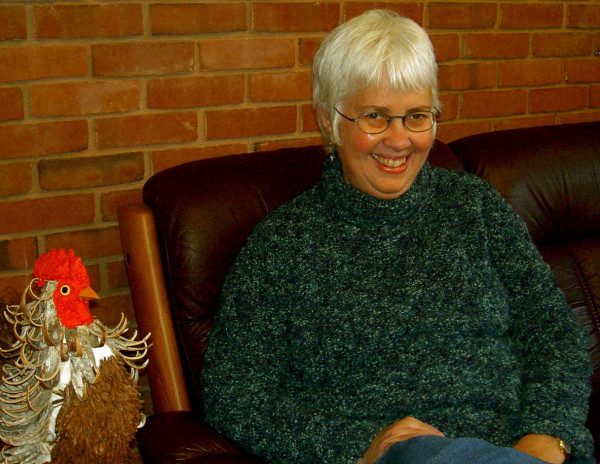
Photograph of Dr. Karen L. Beemon with a model of her favourite animal, a chicken.

Dr. Beemon is Professor and Chair of the Biology Department, Johns Hopkins University. Dr. Beemon received her PhD in 1974 from the University of California, Berkeley, working with Peter Duesberg. She was a postdoctoral fellow at the Salk Institute, working with Tony Hunter. She was among the first to develop and apply molecular techniques to characterize the genomes of RNA viruses, describe recombination between viral genomes, characterize sarcoma-specific sequences, and perform structure-function analysis of Src proteins. Of particular importance, was her discovery that transformation mechanisms of multiple classes of retroviruses involve aberrant phosphorylation of cellular proteins at tyrosine residues. Over the last two decades, Dr. Beemon has contributed significantly to the scientific community's understanding of the role of *cis*-acting regulatory elements in regulation of RNA splicing, polyadenylation, nuclear export and nonsense-mediated RNA decay. Her work has consistently provided a bridge to connect fundamental knowledge of RNA biology to the field of retrovirology. Furthermore, Dr. Beemon's work has revealed the contribution of post-transcriptional gene regulation to viral oncogenesis. Her present studies of insertional events in avian retroviruses are identifying additional new mechanisms that contribute to cancer including activation of microRNA and elevated telomerase expression.

KBL: How did you get interested in retroviruses – was it the vantage point of virology, cell biology, or perhaps the exciting potential of molecular techniques...?

KB: Actually, I wanted to work on messenger RNAs while in graduate school. Since there were not good molecular techniques for specific cellular mRNA analysis at that time, I decided to work on viral RNA instead. Since retroviral packaged genomic RNA is identical to one of the viral mRNAs, the virus helped purify the mRNA for further study. Of course, first we had to characterize the genome. I eventually was able to concentrate on viral mRNA after I got my job at Hopkins.

KBL: Your early research experiences were with well known pioneers of the RNA tumor virus field. What lessons did you learn from them?

KB: My first research mentor at UC Berkeley was Harry Rubin. I learned how to do tissue culture and assays for transformation by Rous sarcoma virus in his lab. I was not very happy with my projects, however, and eventually left his lab and dropped out of graduate school for nearly a year. After travelling and working, I was motivated to go back to graduate school. When I went back, I switched to the lab of Peter Duesberg, where I began working with viral RNA for the first time. We were collaborating with Peter Vogt in trying to determine the complexity of the genome of Rous sarcoma virus. Once we showed that the entire genome was present in one RNA subunit, we started mapping genes, showing that the oncogene src was at the 3' end near the poly(A) sequence. Every 6 weeks or so, we would have joint lab meetings with Peter Vogt's lab at USC, as well as the lab of Michael Bishop and Harold Varmus at UCSF. This was a very exciting time to be working on Rous sarcoma virus and studying the mechanism of viral transformation. Peter Duesberg taught me to pick important problems and to use the best techniques available. He also taught me not to always believe dogma. I moved to the Salk Institute for my postdoc to work with a new PI named Tony Hunter, who allowed me to bring my own research project with me. I was interested in determining the product of the src gene and its mechanism of action. Tony taught me to pay attention to details-that is how he discovered tyrosine phosphorylation. The Salk had a very interactive group of tumor virologists at that time, working with murine leukemia viruses. We also started annual retrovirology meetings with the Fred Hutchinson Cancer Center.

KBL: Your research program remained focused on chicken retroviruses during the progression of many molecular biologists to human retrovirus research. What would you say regarding the scientific funding arena and trend for funding agencies to bypass basic investigations in lieu of "translational" research that more rapidly may move from bench to bedside?

KB: I actually worked on a lentivirus, Visna, while in graduate school-showing that its genomic complexity was the same as that of the RNA tumor viruses. Later, at Hopkins I did some work on HIV-1 splicing, but the general principles found were similar to those previously determined for RSV. I have tried to work on basic questions that were common to all of the retroviruses, such as characterization of the genome and of unspliced RNA. It made sense to me to work with the simplest virus that could give you the answer. I kept finding new questions to try to answer with these viruses, so it didn't seem to make sense to move to a more complex virus until we better understood the simpler viruses. Also, I am interested in how viruses induce cancer.

Since I have mainly undergraduate and graduate students in my lab, it seemed that chicken viruses would be safer for them to work on than HIV-1. Finally, I enjoy being able to do something a little different than everyone else-so that it is not just a race.

I am very disturbed by the current funding climate in which basic science is deemed of lower priority than translational research. Basic research with chicken and other animal viruses led to the discoveries of oncogenes and their mechanism of action. This led to the generation of many drugs currently used in humans, including Gleevec. I think simple systems are much more cost-effective and allow more innovation, resulting in radical changes in therapies. I think a lot of translational research is fairly conservative and does not bring major breakthroughs.

KBL: You have been repeatedly successful in bringing fundamental questions in RNA biology to bear on retrovirus replication and tumorigenesis. What is your advice to aspiring scientists who also seek challenging cross disciplinary work?

KB: I have really enjoyed being at the interface of the RNA and retrovirus fields, especially when the viral RNA behaved differently than predicted by those studying cellular mRNAs. Many of the important discoveries in RNA biology were made by study of viral RNA. The only disadvantage is you have to go to twice as many meetings to keep up with both fields. Also, you have to get used to having your session at the Retroviruses meeting on Sunday morning.

KBL: What is currently the most exciting research in your laboratory?

KB: I am tremendously excited about microRNAs. They seem to play a huge role in oncogenesis, as well as normal development. We currently are studying targets of miR-155, which is activated by ALV promoter insertion in B-cell lymphomas and is up-regulated in many human tumors. Incredibly, this 22 nt RNA is also critical for development of the immune system and inflammatory responses, and even has a homolog in KSHV.

KBL: Your career progression recently evolved to include administration as the first woman to chair the oldest Biology department in the country, which was founded in 1876. Why did you make this change and what is your advice to those considering balancing research and administrative career tracks?

KB: I wanted to be able to shape the direction of the department. In addition, I felt that it was my turn to help administer the department. However, it is very time-consuming and I am glad that I did not take it on earlier in my career.

KBL: Would do you see as your role in mentoring junior faculty and supporting the career development of senior faculty?

KB: I have tried to integrate new junior faculty into the larger Hopkins community so that they would not be isolated scientifically. To this end, I have proposed joint appointments with departments and graduate programs in the School of Medicine. I have also nominated junior faculty to speak at the Cold Spring Harbor Symposium, as well as for various awards. I have also tried to integrate them, gradually, into the service work of the department. Our junior faculty have also developed their own mentoring system and read each other's papers, grants, and promotion packages. I am also trying to develop some Centers to bring people from different schools together. I also encourage junior faculty to attend meetings, write more papers, and help to make the department stronger.

Regarding senior faculty, the challenge is how to use each person's talents most effectively. Some senior faculty would prefer to do research full-time (or part-time) and others would prefer to teach full-time. I am trying to develop mechanisms so this can happen to enhance the careers of the faculty, as well as the overall research and teaching missions of our department.

When I hire new faculty, I am looking for people who love to do research and to teach, but this can change over time. I am also trying to increase the diversity of the department at all levels.

KBL: Academe has been charged as training too many PhDs. What changes do you feel important for training the next generation of successful scientists?

KB: We need to educate our students about careers in addition to academia. However, so far it seems that most of our students are using their Ph.D. training, so it is not clear to me that there are too many Biology Ph.Ds.

KBL: You represent a small cadre of woman who have been promoted to the highest ranks of academe. You have balanced your outstanding research career, administrative service, and an active family life. What is your perspective on the issue of work-life balance?

KB: I am very happy that I was able to have two daughters and a scientific career. For this to work well, I think it is important to have a supportive spouse. Nicole was born while I was a postdoc and told by one PI that I was not allowed to work in the lab while pregnant. However, I ignored him and kept on working without problem. I think that an academic career allows one great flexibility, which is important when you have children. I think that having a family brings some balance to life and also helps in mentoring students. I am very proud of my daughters, even though they are not pursuing careers in science.

KBL: In years to come, how would you like to be remembered?

KB: She loved being a scientist and a mother.
